# Zinc ion dyshomeostasis increases resistance of prostate cancer cells to oxidative stress via upregulation of HIF1α

**DOI:** 10.18632/oncotarget.23893

**Published:** 2018-01-03

**Authors:** David Wetherell, Graham S. Baldwin, Arthur Shulkes, Damien Bolton, Joseph Ischia, Oneel Patel

**Affiliations:** ^1^ Department of Surgery, University of Melbourne, Austin Health, Heidelberg, Victoria, 3084, Australia; ^2^ Department of Urology, Austin Health, Heidelberg, Victoria, 3084, Australia

**Keywords:** castrate resistant, hypoxia inducible factor 1 alpha, prostate cancer, zinc, iron

## Abstract

Zinc ions (Zn^2+^) are known to influence cell survival and proliferation. However the homeostatic regulation of Zn^2+^ and their role in prostate cancer (PC) progression is poorly understood. Therefore the subcellular distribution and uptake of Zn^2+^ in PC cells were investigated. Inductively coupled plasma mass spectroscopy and fluorescent microscopy with the Zn^2+^-specific fluorescent probe FluoZin-3 were used to quantify total and *free* Zn^2+^, respectively, in the normal prostate epithelial cell line (PNT1A) and three human PC cell lines (PC3, DU145 and LNCaP). The effects of Zn^2+^ treatment on proliferation and survival were measured *in vitro* using MTT assays and *in vivo* using mouse xenografts. The ability of Zn^2+^ to protect against oxidative stress via a HIF1α-dependent mechanism was investigated using a HIF1α knock-down PC3 model. Our results demonstrate that the total Zn^2+^ concentration in normal PNT1A and PC cells is similar, but PC3 cells contain significantly higher free Zn^2+^ than PNT1A cells (*p* < 0.01). PNT1A cells can survive better in the presence of high concentrations of Zn^2+^ than PC3 cells. Exposure to 10 µM Zn^2+^ over 72 hours significantly reduces PC3 cell proliferation *in vitro* but not *in vivo*. Zn^2+^ increases PC3 cell survival up to 2.3-fold under oxidative stress, and this protective effect is not seen in PNT1A cells or in a HIF1α-KD PC3 cell model. A state of Zn^2+^ dyshomeostasis exists in PC. HIF1α is an integral component of a Zn^2+^-dependent protective mechanism present in PC3 cells. This pathway may be clinically significant through its contribution to castrate-resistant PC survival.

## INTRODUCTION

The essential trace metal zinc, as the divalent Zn^2+^ ion, is a critical structural component of many proteins including transcription factors, and a component and/or co-factor for > 300 enzymes [1]. The prostate gland contains a very high concentration of zinc ions particularly in the peripheral zone, where most prostate tumours occur. In a normal prostate epithelial cell, zinc ions inhibit the mitochondrial enzyme aconitase (mAC), and consequently citrate accumulates and is secreted at high concentrations into seminal fluid [2].

Compared to the normal prostate, the Zn^2+^ concentration in prostate cancer (PC) is reduced by 80% [3]. Excessive Zn^2+^ is toxic to cells, and therefore regulated homeostasis is critical, but the homeostatic mechanism in PC is poorly understood. Human serum Zn^2+^ concentrations range from 10 to 25µM, most of which is bound to proteins, and the interstitial Zn^2+^ concentration is normally 2 to 5 μM [4]. The majority of Zn^2+^ is tightly bound and considered inactive, and the amount of *free* Zn^2+^, which is considered biologically active, is in the pM to nM range [5]. Unlike most cells in which Zn^2+^ is sequestered into vesicles and organelles, in normal prostate cells 35% of *free* Zn^2+^ in located in the cytoplasm and 30% is sequestered in the mitochondria [6]. The recent development of fluorescent probes specific for the Zn^2+^ ion has made quantifying *free* Zn^2+^ achievable via fluorescent microscopy/spectroscopy, but their application in PC has been limited and little is known about the intracellular *free* Zn^2+^ concentration, Zn^2+^ uptake, or the subcellular distribution of Zn^2+^ in PC cells [7].

Zn^2+^ treatment has been shown to reverse the effects of oxidative stress *in vivo* and to increase resistance to chemo- or radiation-induced apoptosis. Therefore, Zn^2+^ has been implicated in PC survival mechanisms [8]. Hypoxia-inducible factor 1α (HIF1α) forms part of a transcriptional complex which stimulates the expression of > 200 survival genes in response to hypoxia. We have previously demonstrated that overexpression of HIF1α in PC is an independent indicator for PC recurrence, metastatic spread and progression to castration-resistant prostate cancer (CRPC) [9].

The aims of the present study were to measure baseline *free* and total Zn^2+^ concentrations in PC cells and determine the role of Zn^2+^ in the proliferation of prostate cancer cells *in vitro* and *in vivo*. Finally, the ability of Zn^2+^ to protect against oxidative stress, and in particular the role of Zn^2+^ in a HIF1α-dependent mechanism, were investigated.

## RESULTS

### Intracellular distribution of Zn^2+^ is altered in CRPC-like cells

**Figure 1:**
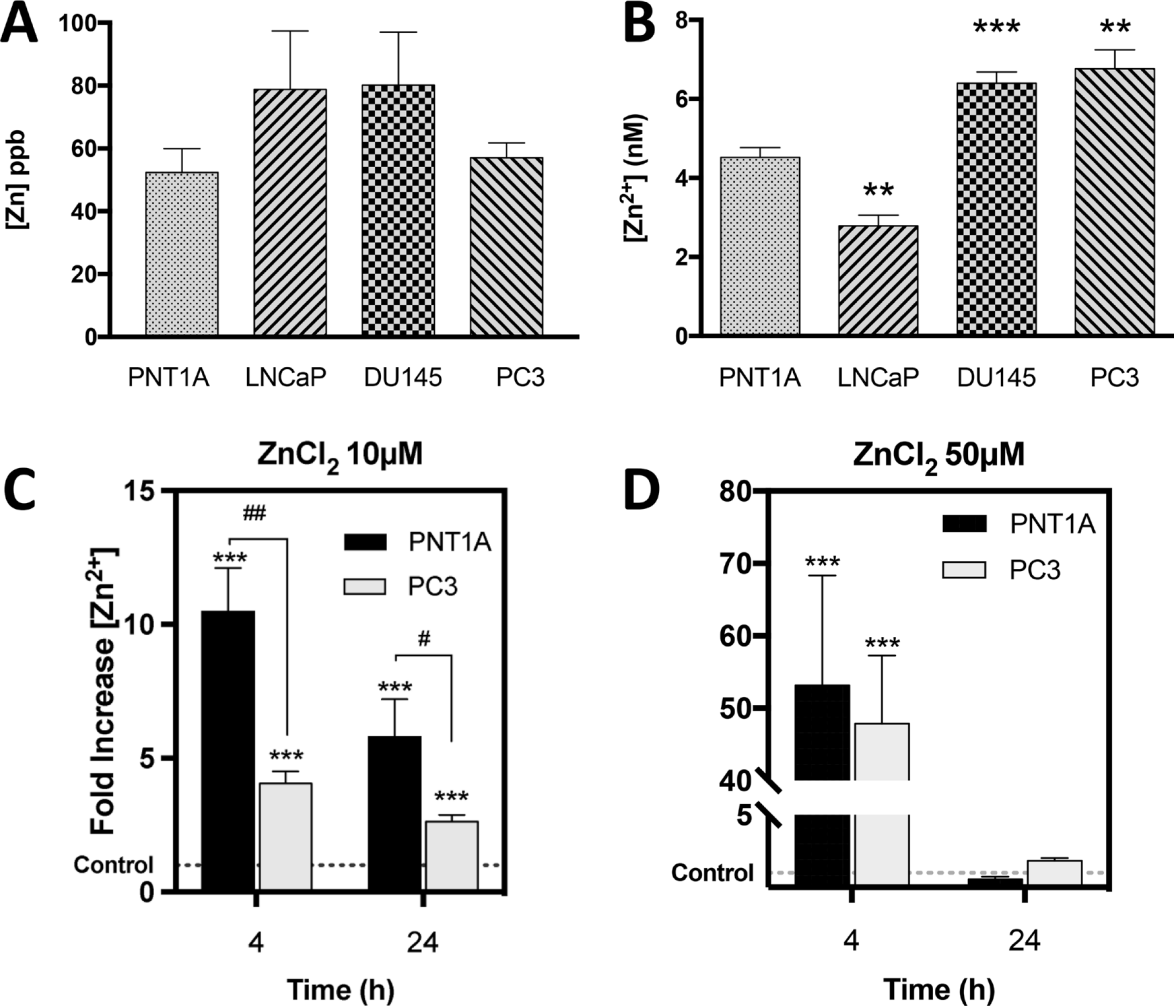
CRPC-like cells contain significantly higher basal free Zn^2+^ ions but equal total zinc compared to normal controls (**A**) Total zinc concentration (ppb) measured by Inductively Coupled Plasma Mass Spectroscopy (ICP-MS) in untreated PNT1A, LNCaP, DU145 and PC3 cells. (**B**) Baseline intracellular *free* zinc (Zn^2+^) concentration (nM) was measured using a FluoZin-3 fluorescent probe in the same 4 prostate cell lines. Zn (nM) = Kd x (F-Fmin)/(Fmax-F) was used to calculate zinc concentration. Intracellular Zn^2+^ uptake following exposure to 10 μM (**C**) or 50 μM (**D**) ZnCl_2_ for 4 or 24 hours was measured in PNT1A and PC3 cells. ^***^p < 0.001 PNT1A vs. PC3 ^##^*p* < 0.05 and ^##^*p* < 0.01. Values are expressed as the mean ± SEM of at least three separate experiments.

Zn^2+^ is abundant in prostate tissue, but cellular Zn^2+^ homeostasis is complex and poorly understood in PC. To address this issue total Zn^2+^ in normal and PC cell lines was measured by Inductively Coupled Plasma Mass Spectroscopy (ICP-MS), which accurately detects Zn^2+^ concentrations as low as 0.5 ppb. Zn^2+^ concentrations (ppb) were 52 ± 8, 79 ± 19, 80 ± 17 and 57 ± 5 for PNT1A, LNCaP, DU145 and PC3 cells, respectively, and there was no significant difference in baseline total Zn^2+^ concentration between normal and PC cells (Figure 1A).

Nearly all intracellular Zn^2+^ ions are tightly bound to proteins and are considered inactive with regard to dynamic biological processes. The very small fraction of *free* Zn^2+^ ions is biologically active and critical to the physiological functions of the cell. A transformation in the pool of *free* Zn^2+^ caused by carcinogenesis could dramatically alter enzymatic reactions and nuclear transcription, thus altering normal cellular functions, including increased survival. Therefore, the concentration (nM) of *free* Zn^2+^ ions was quantified using a fluorescent indicator specific for Zn^2+^ (FluoZin-3) in all four prostate cell lines (Figure 1B). Basal *free* Zn^2+^ concentration (nM) was 4.5 ± 0.2, 2.8 ± 0.3, 6.4 ± 0.3 and 6.8 ± 0.5 in PNT1A, LNCaP, DU145 and PC3 cells, respectively. The CRPC-like PC3 and DU145 cells contained significantly higher, and the androgen-sensitive LNCaP cells significantly lower, *free* Zn^2+^ compared to PNT1A cells (*p* < 0.01).

To rule out the possibility that a difference in Zn^2+^ uptake between PC3 and PNT1A cells could account for the higher *free* Zn^2+^ in PC3 cells, intracellular *free* Zn^2+^ was measured using FluoZin-3 following treatment of both cell types with 10 µM Zn^2+^. Surprisingly *free* Zn^2+^ was actually higher in PNT1A cells than in PC3 cells (Figure 1C). At a higher Zn^2+^ concentration of 50µM, the fold increase in intracellular *free* Zn^2+^ was similar in both cell lines (*p* > 0.05) (Figure 1D). Thus the increased *free* Zn^2+^ in PC3 cells is not due to more rapid Zn^2+^ uptake.

**Figure 2:**
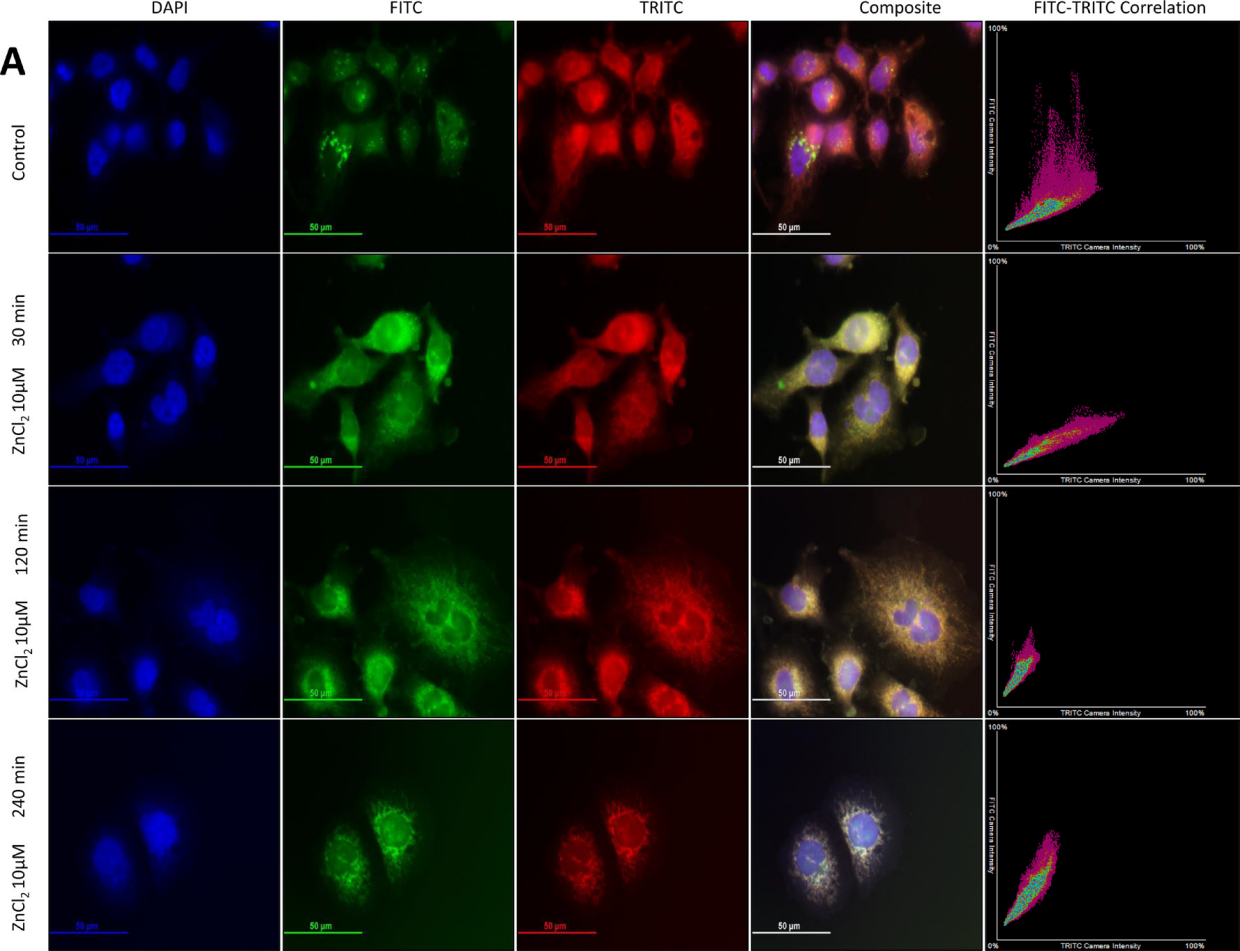

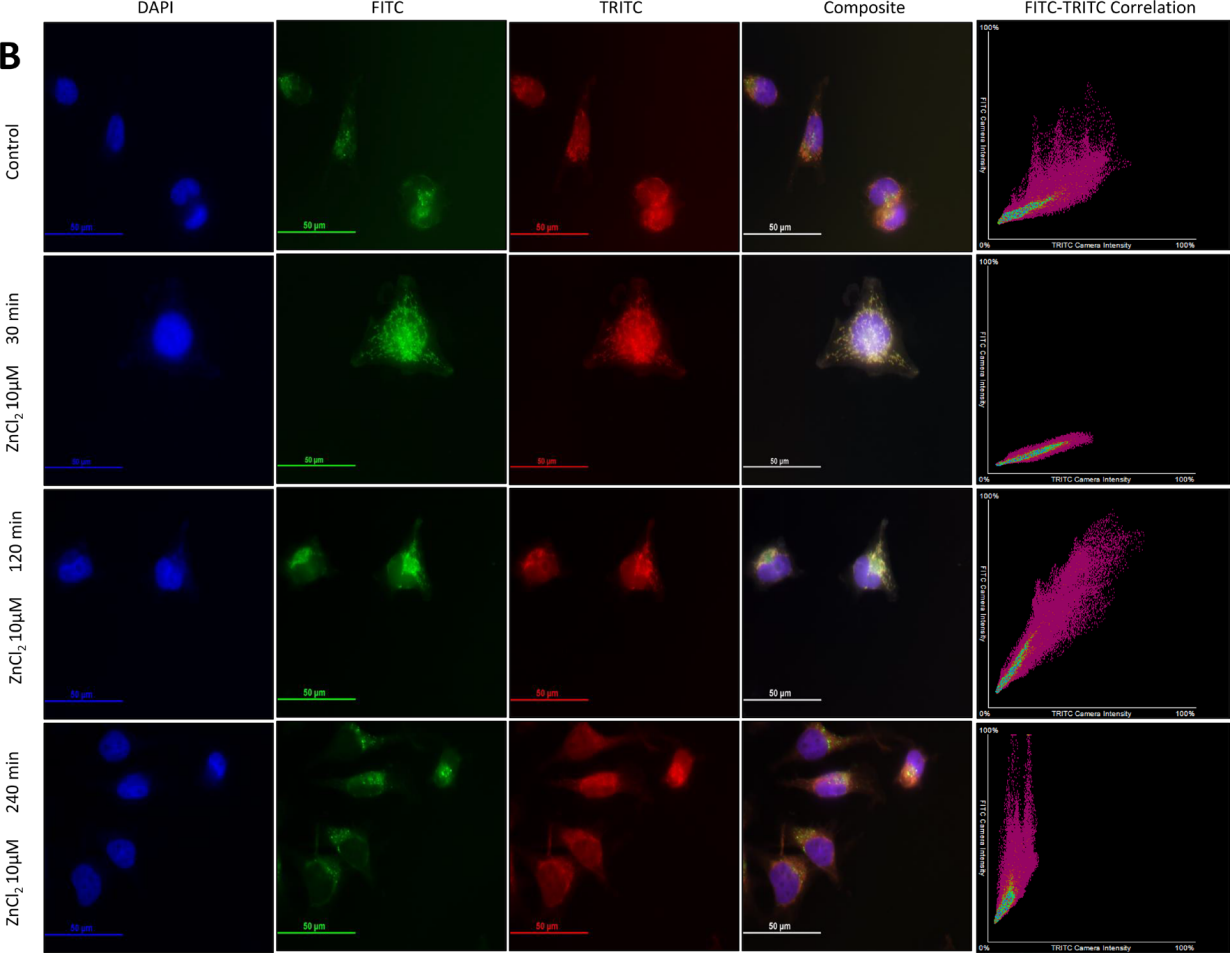

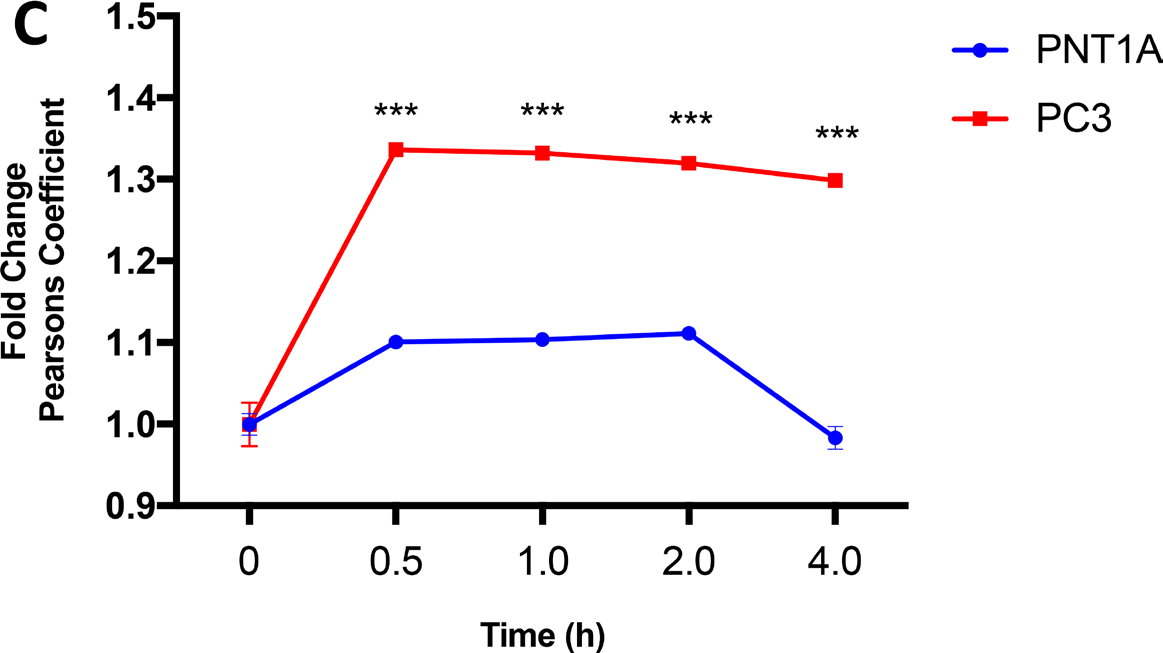
Co-localisation of Zn^2+^ to mitochondria in prostate cells (**A**) PC3 cells and (**B**) PNT1A cells were exposed to 10 μM ZnCl_2_ for 30, 120 and 240 minutes and stained with Hoechst (DAPI), FluoZin-3 (FITC) and MitoTracker (TRITC) fluorescent dyes. Immunofluorescent microscopy images were acquired at 60× (oil immersion) magnification using a Nikon DS-Qi 1Mc Camera with 250 ms, 120 ms and 35 ms exposure times for DAPI, FITC and TRITC channels respectively. Composite images were created by merging blue (DAPI), green (FITC) and red (TRITC) channels. FITC and TRITC colour intensity for each pixel in the corresponding composite image is plotted on scatter plots. (**C**) Co-localisation of Zn^2+^ to mitochondria for PNT1A and PC3 cells was estimated using Pearson correlation coefficient between FITC (FluoZin-3) and TRITC (MitoTracker) colours. Values are expressed as fold change compared to time 0 hours. Statistical significance for the PNT1A vs. PC3 comparison was determined using the Bonferroni-Sidak method with α = 0.05. ^***^p < 0.001. Values are expressed as the mean ± SEM of at least three separate experiments.

To investigate further the disparity in Zn^2+^ homeostasis between PC3 and PNT1A cells, the distribution of Zn^2+^ was evaluated using MitoTracker Red FM (a far red-fluorescent mitochondrial dye) and Hoechst 33342 (a blue nuclear DNA stain) in combination with FluoZin-3 (a green Zn^2+^ indicator). Untreated PC3 cells (Figure 2A) appeared to have larger, distinct intracellular Zn^2+^ pools, which were located more peripherally than in PNT1A cells (Figure 2B). Following exposure to 10µM ZnCl_2_, Zn^2+^ was rapidly (30 min) co-localised to the mitochondria in both cell lines as assessed by coalescence of green and red fluorescence to form yellow. This phenomenon persisted for up to 120 min in PNT1A cells, and beyond 240 min in PC3 cells, before distinct Zn^2+^ pools reappeared similar to the appearance of untreated control cells. The scatter plots in Figure 2A and Figure 2B illustrate the prolonged co-localisation of Zn^2+^ to the mitochondria. The Pearson correlation coefficient (PCC) has been recommended as the gold-standard assessment tool to quantify the degree of co-localisation between two fluorophores [10]. Zn^2+^ was localised to the mitochondria in both the cell lines at 120 min post Zn^2+^ treatment, however at 240 min a clear divergence in behaviour of each cell line was visible with PNT1A cells rapidly returning to the pre-treatment distribution but PC3 cells continuing to localise Zn^2+^ to the mitochondria (*p* < 0.001) (Figure 2C). Thus baseline *free* Zn^2+^, Zn^2+^ uptake and subsequent subcellular distribution of Zn^2+^ in CRPC-like PC cells was strikingly different to normal prostate epithelial cells.

### Exogenous Zn^2+^ is cytotoxic to prostate cells at high doses

**Figure 3:**
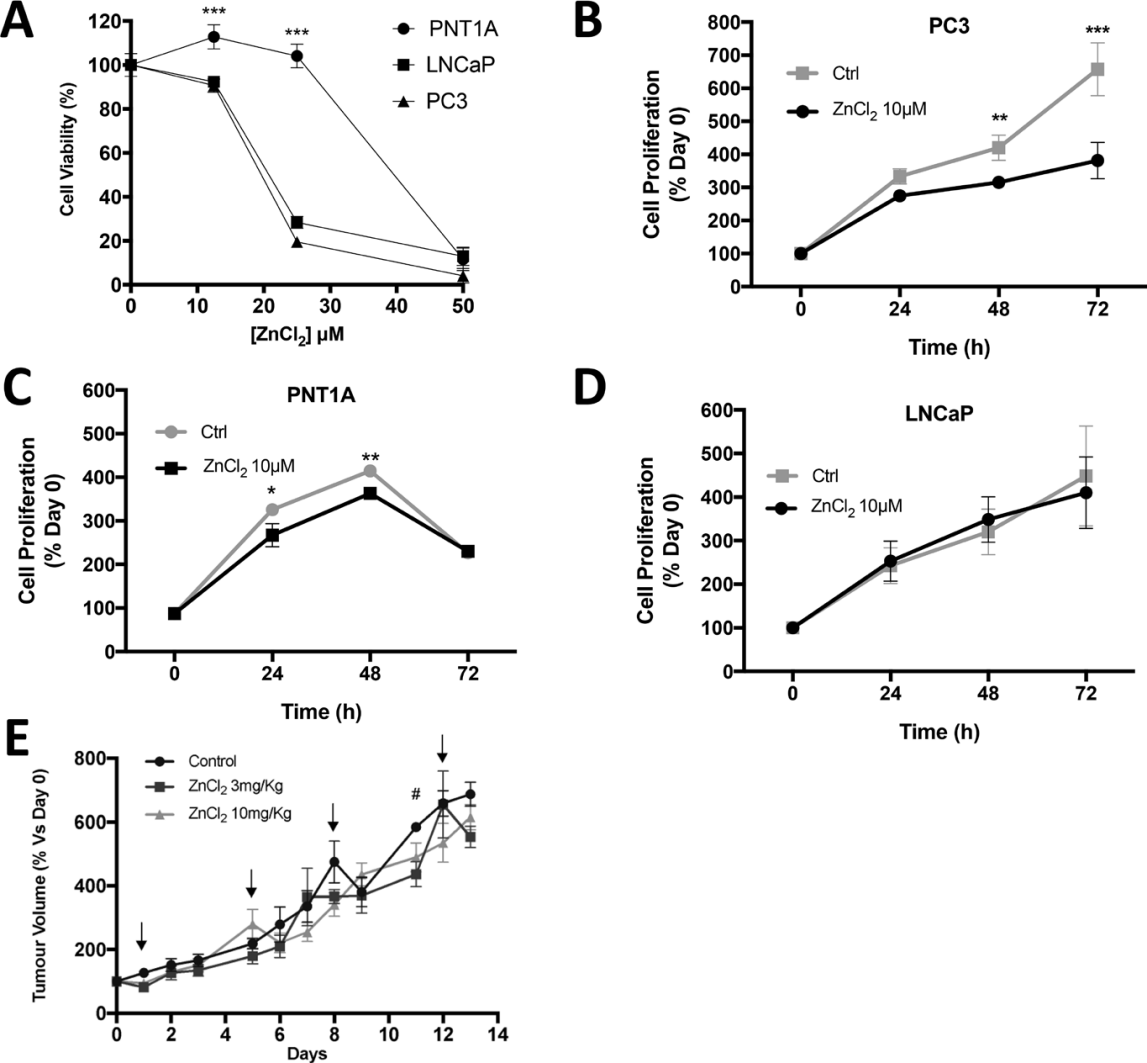
Zinc chloride treatment inhibits *in vitro* cell proliferation in CRPC-like PC3 cells (**A**) Cell viability in PNT1A, LNCaP and PC3 cells after 48 hours of exposure to various concentration of ZnCl_2_ was analysed by MTT assay. Proliferation of cells exposed to serum-free medium only (Ctrl) or to ZnCl_2_ was assessed by MTT assay at 0, 24, 48 and 72 hours respectively in (**B**) PC3, (**C**) PNT1A and (**D**) LNCaP cells. Statistical analysis using one-way ANOVA (Ctrl vs. Zinc) was performed ^*^p < 0.05, ^**^p < 0.01 and ^***^p < 0.001. Values are expressed as the mean ± SEM of at least three separate experiments. (**E**) PC3 xenograft tumour volume in SCID mice in three treatment arms: control (*n* = 7), ZnCl_2_ 3mg/Kg (*n* = 3) and ZnCl_2_ 10mg/Kg (*n* = 7). Arrows represent the days of ZnCl_2_ or saline (control) IP injection. The percentage increase compared to day 0 was calculated and the mean ± SEM plotted. Statistical analysis was calculated by two-way ANOVA. ^#^*p* < 0.05, ZnCl_2_ 3 mg/Kg versus control.

The effect of zinc ions on prostate cell lines was investigated *in vitro* in order to ascertain the sensitivity to Zn^2+^ of normal prostate cells compared to cancer cells. The dose-response of normal prostate (PNT1A), androgen-sensitive (LNCaP) and CRPC-like prostate cancer cells (PC3) to Zn^2+^ (ZnCl_2_) was measured using an MTT cell survival/proliferation assay. All cell lines tolerated exposure to low ZnCl_2_ concentrations (12.5 µM) for 24 hours with 93%, 91% and 113%, of PC3, LNCaP and PNT1A cells surviving, respectively (Figure 3A). However, at 25 µM Zn^2+^ cell survival was 19% in PC3 and 28% in LNCaP compared to 104% in PNT1A cells. Furthermore at a higher concentration (50 µM) Zn^2+^ was cytotoxic to all three cell lines. This observation suggests that normal prostate epithelial cells can survive better in the presence of higher concentrations of Zn^2+^ than prostate cancer cells.

Accumulation and utilisation of Zn^2+^ is a significant property of prostate epithelial cells. We investigated the effect of Zn^2+^ on PC cell proliferation *in vitro* and *in vivo*. A low well tolerated dose of ZnCl_2_ (10 µM) was selected for a long-term (72 hour) MTT cell proliferation assay. At 72 hours relative cell numbers increased 3.8 ± 0.5 fold in Zn^2+^-treated PC3 cells, and the increase was significantly lower than in untreated cells which increased by 6.6 ± 0.8 fold (*p* < 0.001) (Figure 3B). Exposure to Zn^2+^ had a significant effect on proliferation of PNT1A but no effect on LNCaP cells (Figure 3C and 3D). The possibility that serum starvation might affect the viability was confirmed in the case of normal prostate PNT1A cells as viability was decreased at the 72 hr time point under serum starvation either in the presence or absence of zinc (Ctrl). In contrast cancer ous LNCaP and PC3 cells maintained viability over 72 hr.

To supplement the *in vitro* observations of the effects of Zn^2+^ on PC cell proliferation, SCID mice with established PC3 xenograft tumours were injected twice weekly with ZnCl_2_ (3 mg/kg or 10 mg/kg) (Figure 3E). Mice were randomised (up to *n* = 7 per group) to either Zn^2+^-treated or control groups. Tumour volumes (mm^3^) were measured daily with micro-callipers and the values expressed as percentage increase compared to day zero. No significant difference in tumour volume was observed over the 2 week treatment period. In addition, treatment with the Zn^2+^ chelator TPEN at 3 mg/Kg or 10 mg/Kg had no effect on tumour growth. Thus Zn^2+^ does not stimulate or inhibit prostate tumour growth *in vivo*. Furthermore a higher dose of Zn^2+^ (20 mg/Kg) was toxic and all mice in this treatment group were culled after a maximum of 2 doses (data not presented). A higher dose of TPEN (20 mg/Kg) also resulted in toxicity.

### Zn^2+^ augments resistance of PC3 cells to oxidative stress

**Figure 4:**
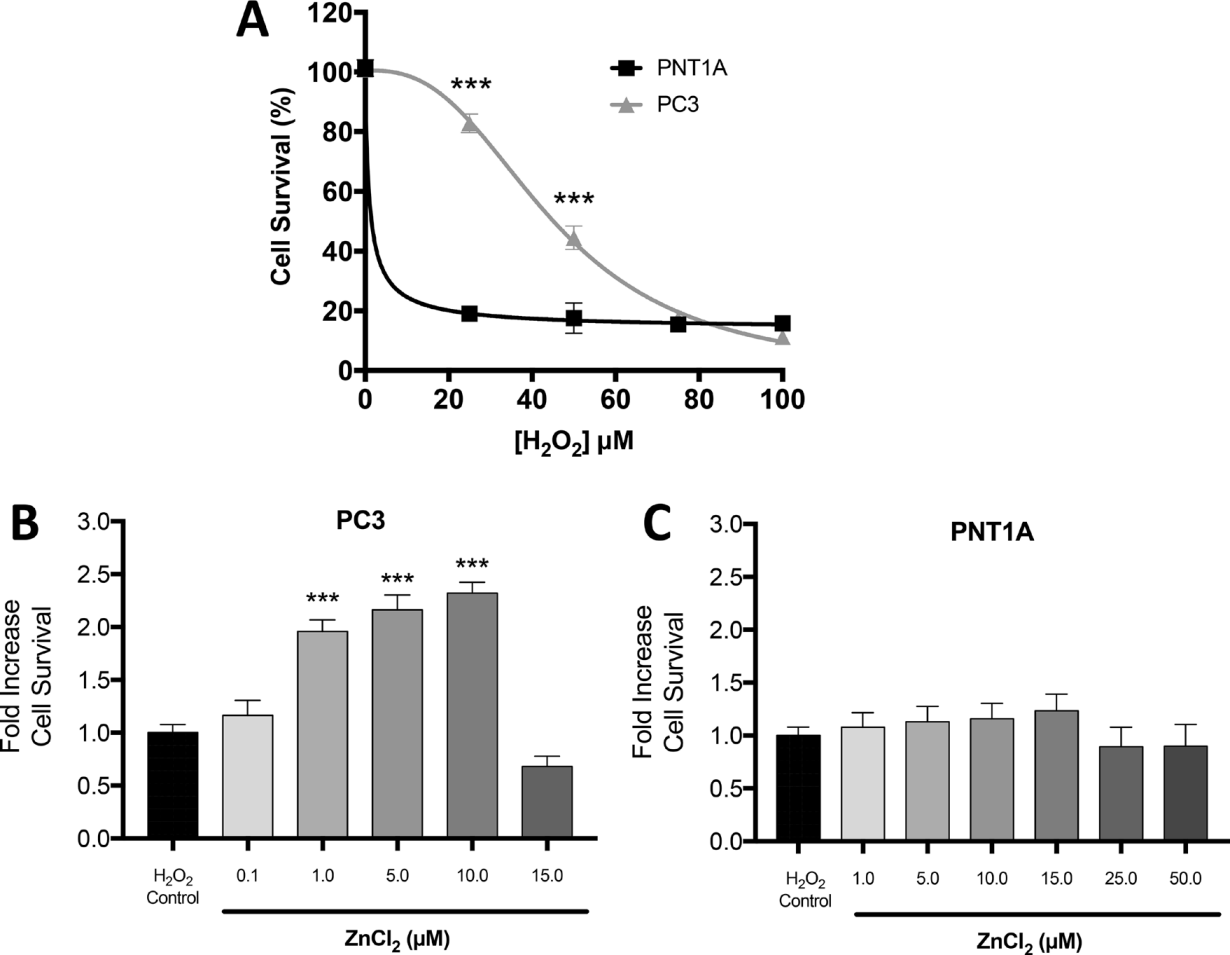
Zn^2+^ mediated protection against oxidative stress injury in PC3 cells (**A**) Cell survival/proliferation was measured by MTT assay. The data demonstrates increased resilience in PC3 cells compared to PNT1A under oxidative stress induced by increasing concentrations of H_2_O_2_. Cell survival was measured by MTT assay in (**B**) PC3 and (**C**) PNT1A cells preconditioned with the indicated concentrations of ZnCl_2_ for 4 hours followed by oxidative stress (75 μM H_2_O_2_ for 24 hours). Values are expressed as the mean ± SEM of at least three separate experiments.

Our previously published study revealed that LNCaP cells, which contain low *free* Zn^2+^, are more sensitive to oxidative stress than PC3 cells, which contain high *free* Zn^2+^ [9]. Therefore, we hypothesized that the higher *free* Zn^2+^ concentration in CPRC-like DU145 and PC3 cells, compared to either normal prostate PNT1A cells or androgen sensitive LNCaP cells, may be responsible for the increased resistance of CRPC-like cells to various stresses. Indeed the basal IC50 value for resistance to H_2_O_2_ was significantly higher at 45.3 µM for PC3 cells compared to 1.8 µM in PNT1A cells (Figure 4A). Furthermore exogenous Zn^2+^ can further increase resistance to oxidative stress by H_2_O_2_ in PC3 cells (with a maximal 2.3 fold greater survival at 10µM Zn^2+^) (Figure 4B), but not in benign PNT1A cells (Figure 4C).

### Zn^2+^ induces HIF1α in prostate cancer cells

**Figure 5:**
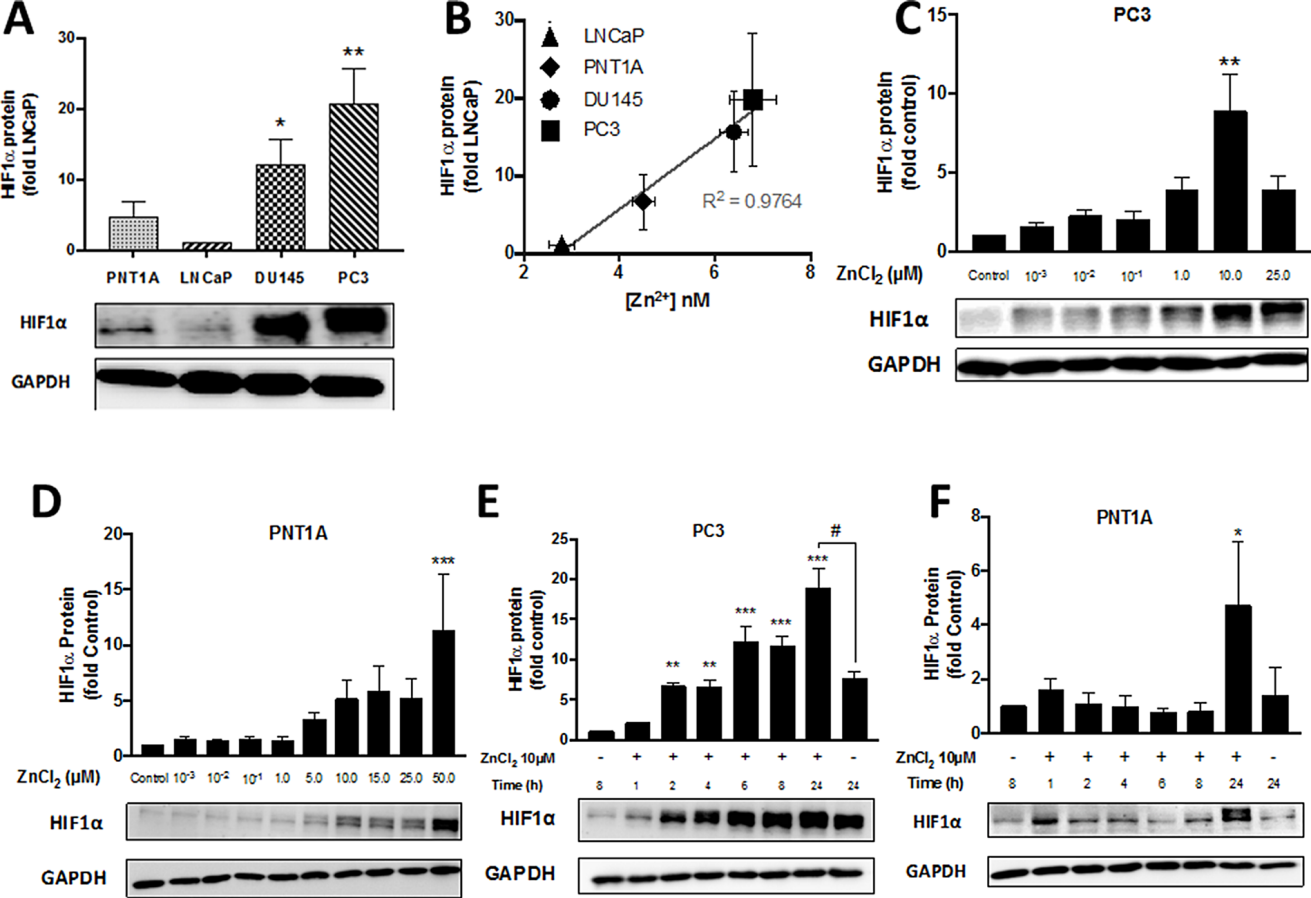
Zn^2+^ induces HIF1α protein expression in PC3 cells in a time- and dose-dependent manner (**A**) Baseline normoxic HIF1α expression is high and (**B**) correlates with *free* Zn^2+^ concentration in PC cells. HIF1α protein expression following treatment with increasing concentrations of ZnCl_2_ for 24 hours was analysed by Western blot in (**C**) PC3 and (**D**) PNT1A cells. Zinc ion-stimulated HIF1α protein expression was measured at the indicated times by Western blot in (**E**) PC3 and (**F**) PNT1A cells. ^*^p < 0.05, ^**^p < 0.01 and ^***^p < 0.001. Values are expressed as the mean ± SEM of at least three separate experiments.

HIF1α is a transcription factor that initiates molecular responses to protect against cellular injury. Expression of HIF1α is upregulated by hypoxia and also by Zn^2+^ ions [11]. We have previously demonstrated that HIF1α is overexpressed in PC3 compared to LNCaP cells under normoxic conditions and that it contributes to resistance to H_2_O_2_ and chemotherapeutics such as 5-fluorouracil [9]. Therefore in this study, the basal expression of HIF1α in PNT1A cells, which contain low concentrations of *free* Zn^2+^, was compared to PC3 cells. Basal expression of HIF1α was significantly greater (21 ± 5 fold) in PC3 cells compared to LNCaP cells (*p* < 0.01) (Figure 5A). Interestingly, normal PNT1A cells expressed up to 4.5 ± 2.3 fold more HIF1α protein than LNCaP cells, and another CRPC-like cell line (DU145) expressed 12.0 ± 3.6 fold higher HIF1α protein. Overall there was a linear correlation (r^2^ = 0.97) between HIF1α expression and concentration of *free* Zn^2+^ in untreated normal prostate epithelial and PC cell lines (Figure 5B). Exogenous Zn^2+^ further increased expression of HIF1α in a dose- and time-dependent manner in PC3 cells, with a maximal increase of 8.9 ± 2.4 fold at 10 µM Zn^2+^ (Figure 5C). Maximal stimulation of HIF1α expression was observed in PNT1A cells at 50 µM Zn^2+^, a concentration which was cytotoxic to PC3 cells (Figure 5D). HIF1α stimulation by Zn^2+^ in PC3 cells was time dependent and occurred as rapidly as 2 h post-exposure (Figure 5E). Increased HIF1α expression was only seen after 24 h in PNT1A cells (Figure 5F).

### Resistance to oxidative stress in PC3 cells is regulated by HIF1α

**Figure 6:**
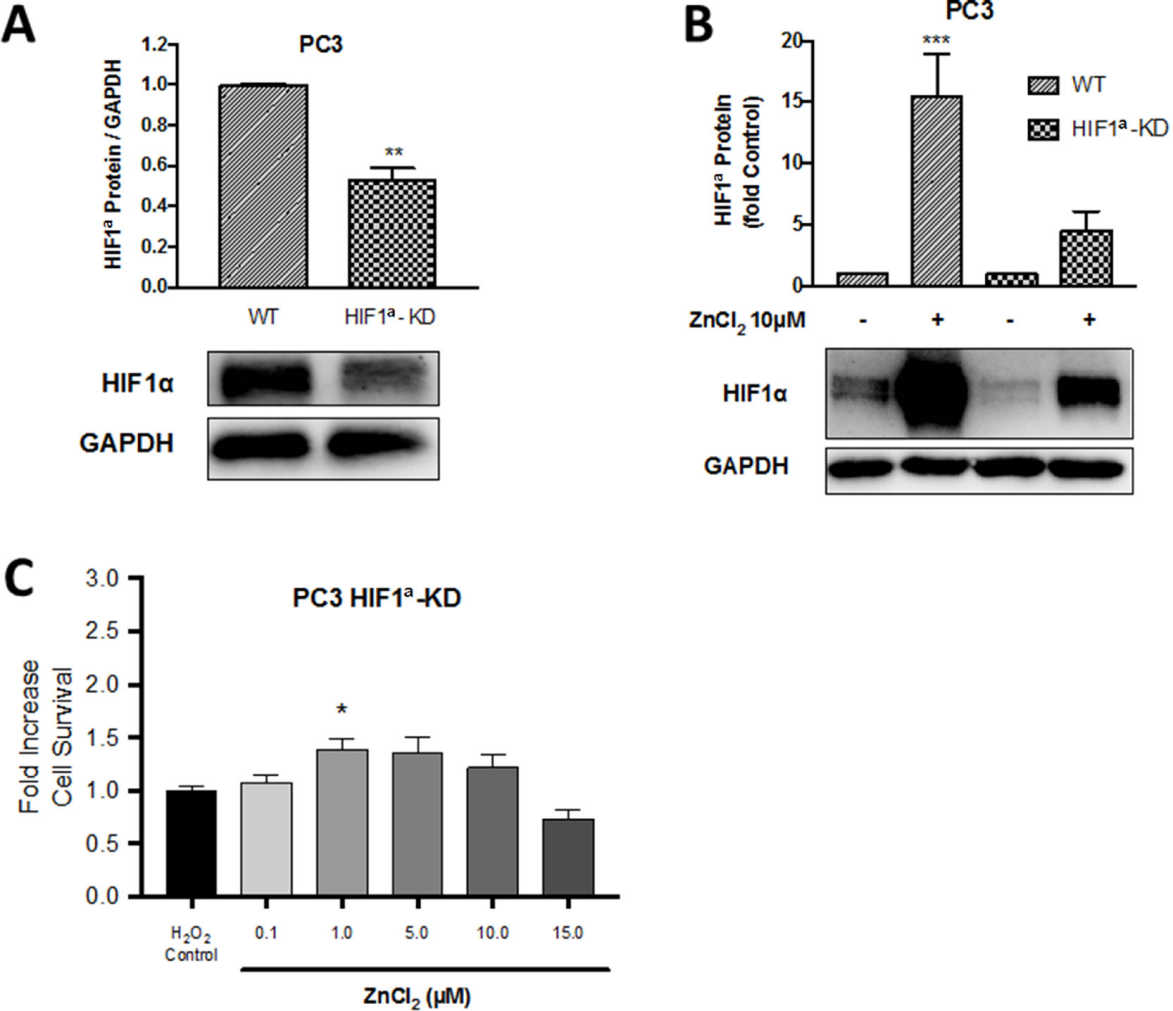
The protective role of Zn^2+^ against oxidative injury in PC3 cells is HIF1α dependent A HIF1α knock-down PC3 cell line (HIF1α-KD) was created by transfection of a shRNA vector expressing HIF1α into wild-type (WT) PC3 cells resulting in (**A**) suppression of HIF1α expression on Western blot. (**B**) HIF1α protein expression in PC3-WT and PC3-HIF1α-KD cells following pre-treatment with 10μM ZnCl_2_ for 4 hours was measured by Western blot. (**C**) Survival/proliferation was measured by MTT assay in HIF1α-KD cells following preconditioning with ZnCl_2_ for 4 hours then exposure to oxidative stress (75 μM H_2_O_2_ for 24 hours). Values are expressed as the mean ± SEM of at least three separate experiments.

Having observed a protective effect of exogenous Zn^2+^ in wild-type PC3 cells, in order to test the hypothesis that this protection was HIF1α dependent, the ability of Zn^2+^ to protect against oxidative stress was examined in a clone of PC3 cells which had been transfected with HIF1α shRNA (HIF1α knock-down or HIF1α-KD cells) [9]. As demonstrated in Figure 6A, HIF1α expression in the HIF1α-KD cell line was reduced by nearly 50% to 0.50 ± 0.06 fold compared to wild-type PC3 cells. Pre-conditioning with ZnCl_2_ (10 µM for 4 hours) significantly increased HIF1α expression by 15.4 ± 3.5 fold in PC3 cells (*p* < 0.001) (Figure 6B). A smaller fold increase in HIF1α expression of 4.5 ± 1.5 was observed in HIF1α-KD PC3 cells. Furthermore, in the HIF1α-KD PC3 cell line a significant protective effect of Zn^2+^ pre-conditioning against oxidative injury was not observed (Figure 6C), unlike the previous results in wild-type PC3 cells (Figure 4B). The reduction in the protection conferred by Zn^2+^ in HIF1α-KD cells implies that HIF1α is an important component of the Zn^2+^-dependent protective mechanism present in PC3 cells. However, as there was some protection still evident in the Zn^2+^-pre-conditioned HIF1α-KD cells, Zn^2+^ ions might be activating alternate mechanisms.

### Zn^2+^ ions competitively inhibit HIF1α degradation by displacing Fe^2+^ ions

**Figure 7:**
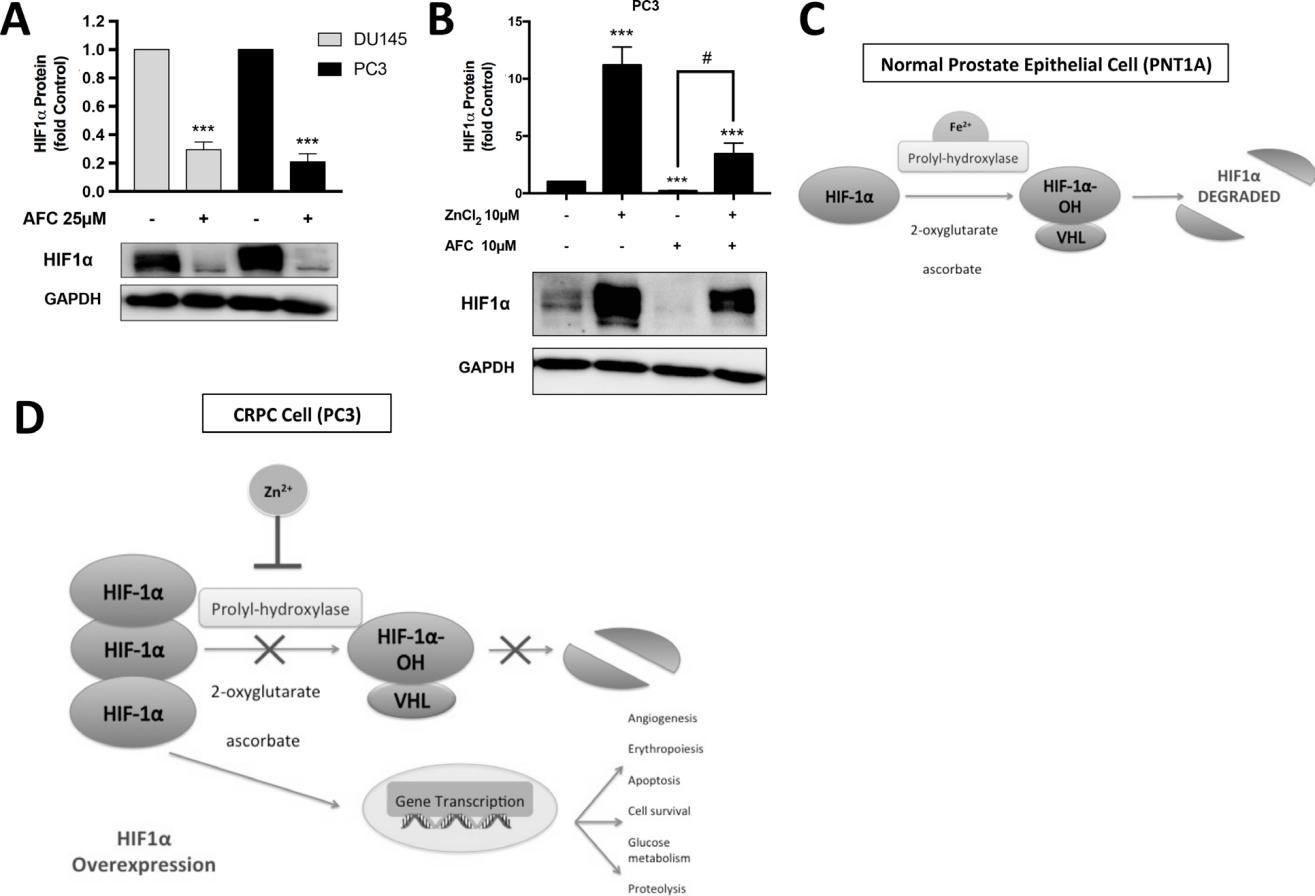
Zn^2+^ ions competitively inhibit HIF1α degradation by displacing Fe^2+^ ions (**A**) HIF1α protein was degraded in the presence of ammonium ferric citrate (AFC) in DU145 and PC3 cells. (**B**) The reduction in HIF1α expression in the presence of iron was partially reversed by Zn^2+^ in PC3 cells. Values are expressed as the mean ± SEM of at least three separate experiments. (**C**) In a normal prostate epithelial cell (PNT1A) under normoxic conditions, the pathway for HIF1α degradation pathway is activated. Proteasomal degradation is achieved by binding to the pVHL-E3-ubiquitin complex mediated by prolyl-hydroxylase (PHD) enzymes which require the co-factors iron (Fe^2+^), ascorbate and 2-oxoglutarate. (**D**) In CRPC cells (PC3) under the same normoxic conditions the HIF1α pathway is inhibited by Zn^2+^ ions, which substitute for Fe^2+^ ions at the PHD binding site, and also potentially reduce the co-factor 2-oxoglutarate via mAC inhibition in the citric acid cycle. Ultimately HIF1α is overexpressed in CRPC leading to increased transcription of genes responsible for glucose metabolism, proteolysis, cell survival, erythropoiesis and angiogenesis.

In many cell types HIF1α is degraded under normoxic conditions by two key sequential processes: firstly the hydroxylation of HIF-α subunits, by prolyl-hydroxylase (PHD) enzymes in an oxygen- and iron (Fe^2+^)-dependent reaction, promotes binding to the pVHL-E3-ubiquitin complex, and secondly HIF1α is destroyed by proteasomal degradation [12]. However, in PC cells under normoxia HIF1α degradation is minimal and there is no decrease in HIF1α translational activity. The exact mechanism is unknown but the available evidence suggests that Zn^2+^ ions compete with Fe^2+^ ions for binding at the active sites of PHDs [13, 14]. In agreement with this hypothesis treatment of CRPC-like cells, which in normoxic conditions over-express HIF1α, with ammonium ferric citrate (AFC) resulted in the degradation of HIF1α to 29% and 20% in DU145 and PC3 cells, respectively, compared to untreated control cells (Figure 7A). More importantly, in the presence of iron, Zn^2+^ rescued HIF1α expression by 3.5 ± 0.95 fold compared to virtually complete degradation of the HIF1α protein in cells treated with AFC alone (*p* < 0.05) (Figure 7B). These observations are consistent with the above hypothesis that Zn^2+^ ions stabilise the HIF1α protein in the presence of oxygen in PC cells by competing with Fe^2+^ ions for binding at the PHD active site (Figure 7D).

## DISCUSSION

Zn^2+^ is essential for cell proliferation and especially for the regulation of DNA synthesis and mitosis, and Zn^2+^ deficiency leads to inhibition of growth [15, 16]. Zn^2+^ stimulates Hep-2 tumour cell proliferation and mitogenic signalling [17], and conversely inhibits proliferation and invasion/migration in some cells including prostate cancer cells [18]. In this study, we have demonstrated that exogenous Zn^2+^ (greater than 15µM) is cytotoxic to prostate cancer cells *in vitro*. Furthermore as normal PNT1A prostate cells had higher IC_50_ values for Zn^2+^ toxicity compared to prostate cancer PC3 cells we concluded that prostate cancer cells are more sensitive to the cytotoxic effect of Zn^2+^ compared to normal cells. In another study, IC_50_ values of Zn^2+^ treatments were 194 μM for non-tumour PNT1A cells and 94 μM for PC3 tumour cells [19]. The discrepancy in IC_50_ values between the two studies may be attributed to the fact that MTT assays in the Masarik *et al.* study were carried out in the presence of FBS which would confer a putative survival advantage [20, 21]. Our results are in agreement with a previous study where CRPC-like DU145 cells were shown to be more sensitive to Zn^2+^ than another normal prostate cancer cell line RWPE-1 [22]. Overall, these observations suggest Zn^2+^ has a cytotoxic effect on prostate cancer cells *in vitro* and, because of this anti-proliferative effect, Zn^2+^ has been hypothesised to be a tumour suppressor in prostate cancer [23].

However, if Zn^2+^ is to be effective as an anti-cancer therapy, its cytotoxic effects need to be observed *in vivo.* Shah *et al.* [24] injected zinc acetate directly into PC3 xenograft tumours and observed a significant reduction in tumour volume. The concentration of Zn^2+^ administered in the Shah *et al.* study [24] equates to 3mM ZnCl_2_, which would immediately be toxic to both normal and cancerous cells, as demonstrated *in vitro* in (Figure 3A). Although intra-tumoural injection in a mouse xenograft tumour model is achievable, the multifocal nature of prostate tumours limits its application in the clinic. Previously a mouse xenograft study using PC3 cells demonstrated that a sustained subcutaneous dosage of zinc sulfate (0.51 mg elemental Zn over 28 days) increased the plasma Zn^2+^ concentration by ∼90%, and resulted in ∼50% inhibition (*P* < 0.05) of tumour growth [25, 26]. In contrast, our results demonstrated that 10 mg/kg ZnCl_2_ (0.4 mg elemental Zn over 14 days) treatment has neither an accelerating nor inhibiting effect on tumour growth (Figure 3E). Previously a single intra-peritoneal injection of 10 mg/kg ZnCl_2_ into SCID mice has been shown to increase *free* intracellular Zn^2+^ and induce gastrin gene expression in colon cancer cells grown as xenograft tumours [21] and two subcutaneous injections of 10 mg/kg ZnCl_2_ into rats protects against renal ischemia reperfusion injury [11]. This suggests that ZnCl_2_ at a10 mg/kg dose is able to stimulate a multitude of biological effects without any toxicity. It could be argued that the 10 mg/kg ZnCl_2_ dose was not enough to cause anti-tumour effects and that a higher Zn^2+^ dose would have reduced tumour growth. However the fact that 20 mg/kg ZnCl_2_ resulted in severe toxicity in mice precludes the use of high doses of Zn^2+^.

Interstitial fluid normally contains 2 to 5µM Zn^2+^, and a cytotoxic effect of Zn^2+^ is observed at concentrations significantly higher than this normal range [24, 27–30]. A lower non-toxic dose of Zn^2+^ did not affect the proliferation of LNCaP prostate cancer cells (Figure 3D). Interestingly Zn^2+^ treatment slowed the proliferation of CRPC-like PC3 cells (Figure 3B) to a much greater degree compared to normal prostate PNT1A cells (Figure 3C). However this was not due to any difference in the uptake of Zn^2+^ because normal PNT1A cells increased intracellular *free* Zn^2+^ equally, rapidly and to a greater degree than PC3 cells exposed to 10 μM ZnCl_2_ (Figure 1C). Furthermore, Masarik *et al* [19] and Kriedt *et al* [28] demonstrated that PC3 cell viability is compromised at earlier Zn^2+^ exposure times and lower doses than low grade LNCaP prostate cancer cells. It seems that an increase in intracellular *free* Zn^2+^ does not necessarily lead to greater proliferation as previously observed [31]. However the observation that the migration of PC3 and DU145 cells, which contain high intracellular *free* Zn^2+^ is high compared to LNCaP cells which contain low *free* Zn^2+^ [9] suggests a role of intracellular Zn^2+^ ions in generation of the non-proliferative but migratory and chemo-resistant phenotype [32] synonymous with CRPC tumors. Overall these results highlight a distinct effect of Zn^2+^ on CRPC-like cells and suggest that the increase in extracellular Zn^2+^ may not be as important as how the cells regulate and cope with a rapid increase in intracellular Zn^2+^. The possibility that Zn^2+^ homeostasis is altered in CRPC-like cancer cells compared to normal cells needs to be investigated.

In human prostate specimens’ total Zn^2+^ concentrations decrease with PC development [2, 33–35]. *In vitro*, although the mean endogenous concentration of Zn^2+^ in LNCaP cells was higher than in PC3 cells, the Zn^2+^ concentrations were not compared to normal prostate cells [36]. A previous study by Qin *et al.* [37] reported a significant reduction in total Zn^2+^ in LNCaP cells compared to normal epithelial RWPE1 cells using ICP-MS. However, the fact that RWPE1 cells were cultured in keratinocyte serum-free medium (KSFM) which contains 0.7 µM Zn^2+^ [38], while LNCaP cells were grown in RPMI which does not contain any detectable amount of Zn^2+^ questions the validity of such results. To overcome these limitations in the current study total Zn^2+^ concentrations were compared by ICP-MS in four prostate cell lines (PNT1A, LNCaP, DU145 and PC3) which were all cultured in the same media (RPMI), and in the absence of any serum which may influence total Zn^2+^ concentrations [20, 21]. The ICP-MS data suggests that there is no correlation between normal and cancerous PC cells *in vitro* in terms of total Zn^2+^ concentrations, although LNCaP cells did contain higher Zn^2+^ than PC3 cells as published previously [36].

In a normal cell, a large proportion of intracellular Zn^2+^ is bound and only a very small proportion is *free*. An increase in intracellular *free* Zn^2+^ ions (the “Zn wave”) may therefore be more relevant in orchestrating downstream biological effects such as increased survival or migration [31, 39]. Interestingly, recent studies have revealed that while total Zn^2+^ is reduced in prostate cancer cells, cytosolic *free* Zn^2+^ is actually higher, emphasizing the importance of measuring *free* Zn^2+^ [37]. Such an increase in *free* cytosolic Zn^2+^ can be attained either by activation of receptors [31, 40, 41] or by increased uptake via Zn^2+^ transporters/channels. Measurement of *free* (also called *labile* or *loosely bound*) Zn^2+^ using the Zn^2+^-specific fluorescent probe FluoZin-3 demonstrated that at baseline CRPC-like PC3 and DU145 cells contained a significantly higher concentration of *free* Zn^2+^ than normal PNT1A cells or low grade androgen-sensitive LNCaP cells. Furthermore calculation of Pearson’s coefficient for the fluorescence microscopy studies indicated that, while both PC3 and PNT1A cells increased Zn^2+^ in the mitochondria in the first 30 min of treatment with exogenous Zn^2+^, only PC3 cells maintained Zn^2+^ in the mitochondria at 4h post initiation of the treatment. It has been suggested that Zn^2+^ may be sequestered in the mitochondria to prevent cytotoxicity [42]. If so, then Zn^2+^ should have been less toxic to PC3 cells compared to PNT1A cells, which was not the case. Overall the hypothesis of Zn^2+^ dys-homeostasis in PC is supported by data from Masarik *et al.* [19] who demonstrated that the free-to-bound Zn^2+^ ratio was much higher in untreated PC3 cells compared to PNT1A cells. Masarik *et al.* also noted that Zn^2+^ was localised in and around nuclei and in the inner part of the cytoplasm in the form of ‘spots’ in PC3 cells which were not seen in PNT1A cells.

Accumulation of high mitochondrial Zn^2+^ concentrations in specific prostate cells results in the induction of apoptosis and the inhibition of cell growth [43]. The opposing notions of the observed ability of Zn^2+^ to be either anti-apoptotic or pro-apoptotic may in part be due to the ability or lack thereof of the mitochondria in different cells to respond directly to the effects of Zn^2+^ [43]. Zn^2+^ ions decrease cell viability and mitochondrial succinate dehydrogenase activity in CRPC-like PC3 cells and benign prostatic hyperplasia epithelial BPH-1 cells [44]. Further mitochondrial Zn^2+^ accumulation has been shown not only to severely impede mitochondrial enzymes such as aconitase, 2-oxoglutarate dehydrogenase, NAD^+^-dependent isocitrate dehydrogenase, and succinate dehydrogenase, but also to induce oxidative stress [45, 46]. These studies raised the possibility that as PC3 cells accumulate greater amounts of Zn^2+^ for a longer period in the mitochondria they may be more sensitive to oxidative stress compared to PNT1A cells. On the contrary, CRPC-like PC3 cells are more resistant to H_2_O_2_ treatment than PNT1A cells, and also exogenous Zn^2+^ at a non-toxic dose can increase further the resistance to oxidative stress. Previously, we have shown that the survival rates of PC3 cells following treatment with either H_2_O_2_ or the chemotherapeutic 5-fluorouracil were significantly greater compared to LNCaP cells via a HIF-dependent mechanism [9].

Hypoxia inducible transcription factors induce diverse genes involved in cell survival, angiogenesis, glucose metabolism and invasion and play an important role in cancer progression [47]. HIF1α degradation normally occurs via oxygen- and iron-dependent prolyl-hydroxylation by prolyl hydroxylase domain-containing protein (PHD) [47]. CRPCs by their nature are extremely resistant to chemotherapy, and we have previously shown an association between HIF1α and resistance to cytotoxic agents [9, 48]. The observation made nearly two decades ago by Zhong and co-workers that PC3 cells overexpress HIF1α even in the presence of oxygen [49, 50] is a very reproducible and robust phenotypic characteristic of CRPC-like cells although the mechanism is unclear. Further the role of Zn^2+^ in HIF1α expression is unclear. Nardinocchi *et al* [51] showed that exogenous Zn^2+^ suppressed both HIF1α and HIF2α protein expression. In contrast, Jeong *et al.* [52] reported a Zn^2+^-mediated dose dependent stimulation of HIF1α protein expression in DU145 and PC3 cells and that HIF1α plays a crucial role in the regulation of zinc resistance [52, 53]. More recently, we have shown that Zn^2+^ induces expression of HIF1α in normal HK-2 renal tubular cells as well as in ACHN renal cancer cells [11]. In this study we have shown a strong correlation between the concentration of *free* Zn^2+^ and HIF1α expression in prostate cells (Figure 5B) but, more importantly, that exogenous Zn^2+^ stimulates HIF1α expression in a time- and dose-dependent manner in PC3 cells with a simultaneous increase in resistance to cytotoxics such as H_2_O_2_ (Figure 4A). Furthermore, as knockdown of HIF1α reduced survival of PC3 cells, even in the presence of Zn^2+^, we concluded that the Zn^2+^-dependent increase in resistance to oxidative stress may in part be via a HIF1α-dependent mechanism in CRPC-like cells.

Our current study using CRPC-like DU145 and PC3 cells confirms previous observation by Knowles *et al* [54] that HIF1α protein expression in reduced in the presence of exogenous iron. More importantly we have establish that exogenous Zn^2+^ was able to reverse the reduction in HIF1α expression in the presence of exogenous iron in PC3 cells (Figure 7B). Overall we postulate that increased *free* Zn^2+^ in CRPC-like PC3 and DU145 cells may displace the Fe^2+^ binding at the PHD site and thereby results in increased stabilization of the HIF1α protein under normal oxygen conditions.

The role of zinc in prostate cancer has been investigated because of changes in the total amount of zinc compared to the normal prostate. Based on the evidence that in prostate cancer the amount of Zn is reduced, it was postulated that re-establishing normal intracellular Zn^2+^ concentrations in prostatic tumours might restore a benign phenotype in malignant prostate cells. However, in contrast Kratochvilova *et al.* and Holubova *et al.* have convincingly showed that exogenous Zn^2+^ drives tumour cells towards a more aggressive and resistant phenotype [53, 55].

In conclusion, a state of Zn^2+^ dys-homeostasis in PC exists as demonstrated by the increased availability and subcellular distribution of *free* Zn^2+^ in PC3 cells. Expression of basal HIF1α correlates with *free* Zn^2+^ concentration in PC cell lines. HIF1α protein can be further increased by exogenous Zn^2+^ in a dose- and time-dependent manner in PC3 cells. Zn^2+^ enhances PC3 cell survival under oxidative stress, an effect not seen in PNT1A cells or in a HIF1α-KD PC3 cell model. Therefore HIF1α is an integral component of a Zn^2+^-dependent protective mechanism in PC3 cells. Based on these findings it can be hypothesized that *free* Zn^2+^ is more relevant especially in CRPC. Furthermore there are no studies which have specifically looked at the role of Zn^2+^ in the chemo-resistant phenotype of CRPC, although the role of HIF1α as a downstream mediator of Zn^2+^ is well established in CRPC [9]. Further studies will be required to establish firstly, if such Zn^2+^ dys-homeostasis may be clinically significant through its contribution to castrate-resistant PC survival and secondly, whether or not Zn^2+^ chelation therapies are effective in counteracting the resistance to treatment of CRPC.

## MATERIALS AND METHODS

### Cell culture and treatment

Normal prostate epithelial cells (PNT1A) were purchased from The European Collection of Cell Cultures (ECACC). Three human prostate cancer cell lines (PC3, DU145 and LNCaP) were purchased from the American Type Culture Collection (Manassas, VA). All cell lines were cultured in Roswell Park Memorial Institute (RPMI) medium (Scoresby, VIC) which was supplemented with 7.5% Fetal Bovine Serum (FBS), 0.4% Penicillin-Streptomycin and 2% HEPES. HIF1α knock-down PC3 cells (HIF1α-KD) were used as previously described [9] .

### Western blot

HIF1α protein was analysed by Western blot using the method previously described [9] with a primary HIF1α purified mouse anti-human antibody at dilution 1:1000 (BD Biosciences, USA) followed by a secondary anti-mouse horseradish peroxidase-conjugated antibody (1:5000, Bio-Rad). GAPDH was assayed as a loading control with a rabbit monoclonal GAPDH antibody (1:10000, Cell Signaling, USA).

### MTT cell proliferation assay

Cells were cultured as described above and trypsinised at 80% confluency. For the MTT cell survival assay cells were plated into a 96-well plate (1.0 × 10^4^ cells/well) and incubated in media containing 7.5% serum for 24 hours. For Zn^2+^ treatment ZnCl_2_ in serum free media (SFM) was added to 12.5 µM, 25 µM or 50 µM, and the cells were incubated for 48 h. For long-term cell proliferation assays cells were plated in four 24-well plates (2.5 × 10^4^ cells/well) representative of each time point (0, 24, 48 and 72 hour) and incubated in SM for 24 h. Next day a MTT assay was performed on one plate to determine the relative cell numbers prior to the start of zinc treatment. The values were recorded as the 0 hr absorbance reading. Cells in the rest of the plates were treated with either 10 µM ZnCl_2_ (10 µM in SFM) or serum free medium only and incubated for 24, 48 or 72 hours. MTT stock solution (5 mg/ml) (Sigma Aldrich, USA) was prepared in 1× PBS and 10 µL/well added. After incubation with MTT solution for 1 h and following the solubilization of formazan crystals in acidified isopropanol the absorbance, which is directly proportional to cell numbers, was measured at a wavelength of 570 nm with background subtraction at 620 nm using a FLUOstar Optima Microplate Reader (BMG Labtech, Mornington, VIC). Data are expressed as a percentage of the 0 hr reading taken just prior to zinc treatment.

### H_2_O_2_ survival assay

Cells were plated on a 12-well plate (1.2 × 10^5^ cells/well) and incubated in culture medium for 24 h. For Zn^2+^ pre-conditioning ZnCl_2_ in SFM was added to the indicated final concentrations between 0.1 µM to 50 µM, and the cells were incubated for 4 h, and the media was then replaced with H_2_O_2_ (25 µM, 50 µM, 75 µM or 100 µM) in SFM for 24 h. MTT analysis was performed as described above.

### FluoZin-3 free Zn^2+^ assay

Cells were plated on a black 96-well plate (1.0 × 10^4^ cells/well) and incubated overnight. Zn^2+^ (10 µM or 50 µM ZnCl_2_ in SFM) was added for 1, 4 or 24 h. 10 µM TPEN or 500 µM ZnCl_2_ were added for 1 h for measurement of Fmin or Fmax, respectively. FluoZin-3 (AM, cell permeant, F-24195, Life Technologies) was added to a final concentration of 5 μM (50 μl/well) and the 96 well plates were covered with foil to protect them from light. Samples were equilibrated for 30 min before the dye was removed and replaced by Hank’s balanced salt solution (HBSS) for 15 min. The resulting fluorescence was recorded on a FLUOstar Optima Microplate Reader (BMG Labtech, Mornington, VIC). Free Zn^2+^ (nM) was calculated according to the manufacturer’s instructions with the formula: Zn^2+^ = Kd × (F-Fmin)/(Fmax-F), where the Kd for FluoZin-3 is 15 nM.

### Fluorescence microscopy (FM)

PNT1A and PC3 cells were mounted onto coverslips overnight then ZnCl_2_ (10 µM in SFM) was added for 30, 60, 120 or 240 min. FluoZin-3 (2.5 µM), MitoTracker Red FM (50nM) or Hoechst 33342 (0.2 µg/mL) was added for 45 min at 37°C in the dark and then fixed by treatment with 4% paraformaldehyde for 3 min. A Nikon DS-Qi 1Mc camera and NIS Nikon Elements Software were used to take separate and composite images, from which mean Pearson correlation coefficient (PCC) values were calculated from 10 images per treatment per cell line.

### Metal analysis by Inductively Coupled Plasma Mass Spectroscopy (ICP-MS)

For measurement of total zinc 5.0 × 10^5^ cells for each cell line (PNT1A, LNCaP, DU145 and PC3) were cultured in 60mm cell culture dishes in 5mls serum media overnight. Next day serum media was aspirated and cells were washed briefly for 10–20 seconds with 2mLs of Milli-Q water. A final volume of 500μL of Milli-Q water was added and cells were scraped and the lysate collected into a 1.5 mL eppendorf tube. Cell lysates were freeze-dried, nitric acid (50 µL of 65%, Suprapur, Merck) was added to each cell pellet, and the pellets were digested overnight at room temperature. The samples were heated using a heating block at 90°C for 20 min to a volume of ∼40 µL. To each sample 460µL of 1% (v/v) of nitric acid diluent was added to a final Volume of 0.5 mL. Measurements were made using an Agilent 7700 series ICP-MS instrument under routine multi-element operating conditions using a helium reaction gas cell. The instrument was calibrated using 0, 5, 10, 50, 100 and 500 ppb of certified multi- element ICP-MS standard calibration solutions (ICP-MS-CAL2-1, ICP-MS-CAL-3 and ICP-MS-CAL-4, Accustandard) for a range of elements. A certified internal standard solution containing 200 ppb of Yttrium (Y89) was used as an internal control (ICP- MS-IS-MIX1-1, Accustandard).

### *In vivo* study

The animal studies (project number A2014/05210) were approved by the Animal Ethics Committees of Austin Health, Victoria, Australia, in accordance with the guidelines laid down by the National Health and Medical Research Council of Australia’s Code of Practice for the Care and Use of Animals for Experimental Purposes. Certified severe combined immunodeficiency (SCID) male mice, aged > 4 weeks were purchased from the Animal Resource Centre (Perth, Australia) and housed in the BioResources Facility (Austin Health). PC3 cells were injected into the flanks of the mice and xenograft tumours were allowed to develop. Tumour volumes were measured daily and mice were randomised to treatment or control groups when the tumour volume was > 200 mm^3^. Treatment consisted of twice-weekly intra-peritoneal injections of ZnCl_2_ or the Zn^2+^ chelator TPEN (Sigma-Aldrich, Australia) (3 mg/Kg, 10 mg/Kg or 20 mg/Kg) until the tumour volume exceeded 1000 mm^3^. Xenograft tissue was harvested for immunohistochemistry (IHC) and Zn^2+^ analysis. Zinc chloride was dissolved in 0.1% HCL in injection water and diluted to a final concentration of 1 μg/μL in saline. TPEN (Sigma-Aldrich) was dissolved in DMSO and diluted with saline to a final concentration of 1 μg/μL. The final DMSO concentration was < 4.0 %.

### Data analysis

Statistics were analysed with GraphPad Prism (Version 7). All experiments were repeated in triplicate. Values are expressed as mean ± standard error of the mean (SEM). Statistical significance for single comparisons of normally distributed data was determined by a two-way Student’s *t* test. For multiple comparisons, one-way ANOVAs followed by the Bonferroni correction were performed. Statistical significance was determined by *p* value < 0.05 (borderline significant), *p* < 0.01 (moderately significant) and *p* < 0.001 (strongly significant).

## References

[1] Fukada T, Yamasaki S, Nishida K, Murakami M, Hirano T (2011). Zinc homeostasis and signaling in health and diseases: Zinc signaling. J Biol Inorg Chem.

[2] Costello LC, Franklin RB (1998). Novel role of zinc in the regulation of prostate citrate metabolism and its implications in prostate cancer. Prostate.

[3] Costello LC, Franklin RB (2011). Zinc is decreased in prostate cancer: an established relationship of prostate cancer!. J Biol Inorg Chem.

[4] Reyes JG (1996). Zinc transport in mammalian cells. Am J Physiol.

[5] Franklin RB, Costello LC (2009). The Important Role of the Apoptotic Effects of Zinc in the Development of Cancers. J Cell Biochem.

[6] Kelleher SL, McCormick NH, Velasquez V, Lopez V (2011). Zinc in specialized secretory tissues: roles in the pancreas, prostate, and mammary gland. Adv Nutr.

[7] Maret W (2015). Analyzing free zinc(II) ion concentrations in cell biology with fluorescent chelating molecules. Metallomics.

[8] Smith DJ, Jaggi M, Zhang W, Galich A, Du C, Sterrett SP, Smith LM, Balaji KC (2006). Metallothioneins and resistance to cisplatin and radiation in prostate cancer. Urology.

[9] Ranasinghe WK, Xiao L, Kovac S, Chang M, Michiels C, Bolton D, Shulkes A, Baldwin GS, Patel O (2013). The role of hypoxia-inducible factor 1alpha in determining the properties of castrate-resistant prostate cancers. PLoS ONE.

[10] Adler J, Parmryd I (2010). Quantifying colocalization by correlation: the Pearson correlation coefficient is superior to the Mander’s overlap coefficient. Cytometry A.

[11] Rao K, Sethi K, Ischia J, Gibson L, Galea L, Xiao L, Yim M, Chang M, Papa N, Bolton D, Shulkes A, Baldwin GS, Patel O (2017). Protective effect of zinc preconditioning against renal ischemia reperfusion injury is dose dependent. PLoS ONE.

[12] Haase VH (2006). The VHL/HIF oxygen-sensing pathway and its relevance to kidney disease. Kidney Int.

[13] Kaczmarek M, Cachau RE, Topol IA, Kasprzak KS, Ghio A, Salnikow K (2009). Metal ions-stimulated iron oxidation in hydroxylases facilitates stabilization of HIF-1 alpha protein. Toxicol Sci.

[14] Li Q, Chen H, Huang X, Costa M (2006). Effects of 12 metal ions on iron regulatory protein 1 (IRP-1) and hypoxia-inducible factor-1 alpha (HIF-1alpha) and HIF-regulated genes. Toxicol Appl Pharmacol.

[15] Beyersmann D, Haase H (2001). Functions of zinc in signaling, proliferation and differentiation of mammalian cells. Biometals.

[16] MacDonald RS (2000). The role of zinc in growth and cell proliferation. J Nutr.

[17] Rudolf E, Cervinka M (2008). External zinc stimulates proliferation of tumor Hep-2 cells by active modulation of key signaling pathways. J Trace Elem Med Biol.

[18] Liang JY, Liu YY, Zou J, Franklin RB, Costello LC, Feng P (1999). Inhibitory effect of zinc on human prostatic carcinoma cell growth. Prostate.

[19] Masarik M, Gumulec J, Hlavna M, Sztalmachova M, Babula P, Raudenska M, Pavkova-Goldbergova M, Cernei N, Sochor J, Zitka O, Ruttkay-Nedecky B, Krizkova S, Adam V (2012). Monitoring of the prostate tumour cells redox state and real-time proliferation by novel biophysical techniques and fluorescent staining. Integr Biol.

[20] Haase H, Hebel S, Engelhardt G, Rink L (2015). The biochemical effects of extracellular Zn(2+) and other metal ions are severely affected by their speciation in cell culture media. Metallomics.

[21] Marshall KM, Laval M, Estacio O, Hudson DF, Kalitsis P, Shulkes A, Baldwin GS, Patel O (2015). Activation by zinc of the human gastrin gene promoter in colon cancer cells *in vitro* and *in vivo*. Metallomics.

[22] Hong SH, Choi YS, Cho HJ, Lee JY, Kim JC, Hwang TK, Kim SW (2012). Antiproliferative effects of zinc-citrate compound on hormone refractory prostate cancer. Chin J Cancer Res.

[23] Franklin RB, Costello LC (2007). Zinc as an anti-tumor agent in prostate cancer and in other cancers. Arch Biochem Biophys.

[24] Shah MR, Kriedt CL, Lents NH, Hoyer MK, Jamaluddin N, Klein C, Baldassare J (2009). Direct intra-tumoral injection of zinc-acetate halts tumor growth in a xenograft model of prostate cancer. J Exp Clin Cancer Res.

[25] Feng P, Li TL, Guan ZX, Franklin RB, Costello LC (2003). Effect of zinc on prostatic tumorigenicity in nude mice. Ann N Y Acad Sci.

[26] Costello LC, Franklin RB (2017). Decreased zinc in the development and progression of malignancy: an important common relationship and potential for prevention and treatment of carcinomas. Expert Opin Ther Targets.

[27] Magneson GR, Puvathingal JM, Ray WJ (1987). The concentrations of free Mg2+ and free Zn2+ in equine blood plasma. J Biol Chem.

[28] Kriedt CL, Baldassare J, Shah M, Klein C (2010). Zinc functions as a cytotoxic agent for prostate cancer cells independent of culture and growth conditions. J Exp Ther Oncol.

[29] Hasumi M, Suzuki K, Matsui H, Koike H, Ito K, Yamanaka H (2003). Regulation of metallothionein and zinc transporter expression in human prostate cancer cells and tissues. Cancer Lett.

[30] Tsui KH, Chang PL, Juang HH (2006). Zinc blocks gene expression of mitochondrial aconitase in human prostatic carcinoma cells. Int J Cancer.

[31] Chang M, Xiao L, Shulkes A, Baldwin GS, Patel O (2016). Zinc Ions Mediate Gastrin Expression, Proliferation, and Migration Downstream of the Cholecystokinin-2 Receptor. Endocrinology.

[32] Yano S, Miwa S, Mii S, Hiroshima Y, Uehara F, Yamamoto M, Kishimoto H, Tazawa H, Bouvet M, Fujiwara T, Hoffman RM (2014). Invading cancer cells are predominantly in G0/G1 resulting in chemoresistance demonstrated by real-time FUCCI imaging. Cell Cycle.

[33] Zaichick VY, Sviridova TV, Zaichick SV (1997). Zinc in the human prostate gland: Normal, hyperplastic and cancerous. Int Urol Nephrol.

[34] Kerr WK, Keresteci AG, Mayoh H (1960). The distribution of zinc within the human prostate. Cancer.

[35] Gumulec J, Masarik M, Adam V, Eckschlager T, Provaznik I, Kizek R (2014). Serum and tissue zinc in epithelial malignancies: a meta-analysis. PLoS ONE.

[36] Costello LC, Liu Y, Zou J, Franklin RB (1999). Evidence for a zinc uptake transporter in human prostate cancer cells which is regulated by prolactin and testosterone. J Biol Chem.

[37] Qin Y, Miranda JG, Stoddard CI, Dean KM, Galati DF, Palmer AE (2013). Direct comparison of a genetically encoded sensor and small molecule indicator: implications for quantification of cytosolic Zn(2+). ACS Chem Biol.

[38] Huang L, Kirschke CP, Zhang Y (2006). Decreased intracellular zinc in human tumorigenic prostate epithelial cells: a possible role in prostate cancer progression. Cancer cell international.

[39] Maret W (2013). Zinc biochemistry: from a single zinc enzyme to a key element of life. Adv Nutr.

[40] Sanchez-Blazquez P, Rodriguez-Munoz M, Bailon C, Garzon J (2012). GPCRs promote the release of zinc ions mediated by nNOS/NO and the redox transducer RGSZ2 protein. Antioxid Redox Signal.

[41] Kaltenberg J, Plum LM, Ober-Blobaum JL, Honscheid A, Rink L, Haase H (2010). Zinc signals promote IL-2-dependent proliferation of T cells. Eur J Immunol.

[42] Lu Q, Haragopal H, Slepchenko KG, Stork C, Li YV (2016). Intracellular zinc distribution in mitochondria, ER and the Golgi apparatus. Int J Physiol Pathophysiol Pharmacol.

[43] Feng P, Liang JY, Li TL, Guan ZX, Zou J, Franklin RB, Costello LC (2000). Zinc induces mitochondria apoptogenesis in prostate cells. Mol Urol.

[44] Untergasser G, Rumpold H, Plas E, Witkowski M, Pfister G, Berger P (2000). High levels of zinc ions induce loss of mitochondrial potential and degradation of antiapoptotic Bcl-2 protein in *in vitro* cultivated human prostate epithelial cells. Biochem Biophys Res Commun.

[45] Lemire J, Mailloux R, Appanna VD (2008). Zinc toxicity alters mitochondrial metabolism and leads to decreased ATP production in hepatocytes. J Appl Toxicol.

[46] McCord MC, Aizenman E (2014). The role of intracellular zinc release in aging, oxidative stress, and Alzheimer’s disease. Front Aging Neurosci.

[47] Ranasinghe WK, Baldwin GS, Bolton D, Shulkes A, Ischia J, Patel O (2015). HIF1alpha expression under normoxia in prostate cancer--which pathways to target?. J Urol.

[48] Tannock IF, de Wit R, Berry WR, Horti J, Pluzanska A, Chi KN, Oudard S, Theodore C, James ND, Turesson I, Rosenthal MA, Eisenberger MA, Investigators TAX (2004). Docetaxel plus prednisone or mitoxantrone plus prednisone for advanced prostate cancer. N Engl J Med.

[49] Zhong H, Agani F, Baccala AA, Laughner E, Rioseco-Camacho N, Isaacs WB, Simons JW, Semenza GL (1998). Increased expression of hypoxia inducible factor-1alpha in rat and human prostate cancer. Cancer Res.

[50] Zhong H, Chiles K, Feldser D, Laughner E, Hanrahan C, Georgescu MM, Simons JW, Semenza GL (2000). Modulation of hypoxia-inducible factor 1alpha expression by the epidermal growth factor/phosphatidylinositol 3-kinase/PTEN/AKT/FRAP pathway in human prostate cancer cells: implications for tumor angiogenesis and therapeutics. Cancer Res.

[51] Nardinocchi L, Pantisano V, Puca R, Porru M, Aiello A, Grasselli A, Leonetti C, Safran M, Rechavi G, Givol D, Farsetti A, D’Orazi G (2010). Zinc downregulates HIF-1α and inhibits its activity in tumor cells *in vitro* and *in vivo*. PLoS ONE.

[52] Jeong CW, Yoon CY, Jeong SJ, Hong SK, Byun SS, Kwak C, Lee SE (2013). The Role of Hypoxia-Inducible Factor-1alpha and -2alpha in Androgen Insensitive Prostate Cancer Cells. Urol Oncol.

[53] Holubova M, Axmanova M, Gumulec J, Raudenska M, Sztalmachova M, Babula P, Adam V, Kizek R, Masarik M (2014). KRAS NF-kappaB is involved in the development of zinc resistance and reduced curability in prostate cancer. Metallomics.

[54] Knowles HJ, Raval RR, Harris AL, Ratcliffe PJ (2003). Effect of ascorbate on the activity of hypoxia-inducible factor in cancer cells. Cancer Res.

[55] Kratochvilova M, Raudenska M, Heger Z, Richtera L, Cernei N, Adam V, Babula P, Novakova M, Masarik M, Gumulec J (2017). Amino Acid Profiling of Zinc Resistant Prostate Cancer Cell Lines: Associations With Cancer Progression. Prostate.

